# One-stage hybrid coronary revascularization for the treatment of multivessel coronary artery disease— Periprocedural and long-term results from the “HYBRID-COR” feasibility study

**DOI:** 10.3389/fcvm.2022.1016255

**Published:** 2022-10-19

**Authors:** Krzysztof Sanetra, Piotr Paweł Buszman, Justyna Jankowska-Sanetra, Marek Cisowski, Wojciech Fil, Bogdan Gorycki, Andrzej Bochenek, Monika Slabon-Turska, Marta Konopko, Paweł Kaźmierczak, Witold Gerber, Krzysztof Milewski, Paweł Eugeniusz Buszman

**Affiliations:** ^1^Clinic of Cardiovascular Surgery, Andrzej Frycz Modrzewski Krakow University, Kraków, Poland; ^2^Department of Cardiac Surgery, American Heart of Poland, Bielsko-Biała, Poland; ^3^Department of Cardiology, Andrzej Frycz Modrzewski Krakow University, Kraków, Poland; ^4^Department of Cardiology, American Heart of Poland, Bielsko-Biała, Poland; ^5^Center for Cardiovascular Research and Development, American Heart of Poland, Katowice, Poland; ^6^Department of Cardiac Surgery, University Hospital, Institute of Medical Sciences, University of Opole, Opole, Poland; ^7^Faculty of Medicine, University of Technology, Katowice, Poland; ^8^Department of Obstetrics and Gynecology, Provincial Specialist Hospital, Wrocław, Poland; ^9^American Heart of Poland, Katowice, Poland; ^10^Department of Epidemiology, Medical University of Silesia, Katowice, Poland

**Keywords:** coronary artery disease, EACAB, hybrid, revascularization, one-stage

## Abstract

**Background:**

The constant growth of interest in hybrid coronary artery revascularization (HCR) is apparent. Yet, few studies report outcomes of the one-stage HCR. Consequently, the status of such procedures is not adequately supported in clinical guidelines. The aim of this study was to report the safety, feasibility, and long term-outcomes of the one-stage HCR.

**Methods and results:**

Patients were enrolled in the prospective one-stage hybrid coronary revascularization program (HYBRID-COR). They underwent a one-stage hybrid revascularization procedure while on double antiplatelet therapy (DAPT) with Ticagrelor: endoscopic atraumatic coronary artery bypass grafting (EACAB) for revascularization of the left anterior descending (LAD) artery and percutaneous intervention in non-LAD arteries with contemporary drug-eluting stents. The composite primary endpoint included MACCE (major adverse cardiac and cerebrovascular events: death, myocardial infarction, stroke, and repeated revascularization) in long-term observation. The study cohort consisted of 30 patients (68% male) with stable coronary artery disease (26.7%) and unstable angina (73.3%). Procedural success was 100%. No death, myocardial infarction (MI), or stroke were observed in the perioperative period. One patient (3.3%) required chest revision and blood transfusion due to surgical bleeding. Kidney injury was noted in two patients (6.6%). In a long-term follow-up (median; IQR: 4.25; 2.62–4.69 years), two patients (6.6%) underwent repeated revascularization and one patient (3.3%) died due to MI. The overall primary endpoint rate was 9.9%.

**Conclusion:**

One-stage hybrid revascularization, on DAPT, is a feasible, safe, and efficient way of achieving complete revascularization in selected patients. The complication rate is low and acceptable. Further randomized trials are required.

## Introduction

Hybrid treatment for coronary artery disease, defined as a mini-invasive, sternal-sparing surgical procedure of LITA-LAD (left internal thoracic artery–left anterior descending artery) grafting combined with percutaneous intervention in non-LAD arteries with the use of contemporary drug eluting stents (DES), is an innovative treatment, performed in experienced cardiovascular centers. However, there are no clear guidelines regarding patient selection, procedure protocol, and time frames for surgical and percutaneous interventions.

According to clinical guidelines, coronary artery bypass grafting remains a standard treatment for multivessel coronary artery disease, especially LITA-LAD grafting (1). However, new generation DES provides satisfactory short- and long-term clinical outcomes, comparable to or superior to saphenous vein grafts (2, 3).

There are few randomized trials on hybrid coronary treatment vs. coronary artery bypass grafting. In a randomized trial of 200 patients, 1- and 5-year rates of death, MI, stroke, and major bleeding or repeat revascularization were not significantly different between hybrid revascularization and CABG (4, 5). However, it must be noted that hybrid revascularization protocol is not unified and has numerous variations in different departments.

First of all, the optimal staging of hybrid procedures remains a matter of debate, mainly due to optimization and timing of administration of a double anti-platelet therapy. Each strategy has its drawbacks and the superiority of one over another is not well established. The randomized trial referenced above provides information only on two-stage hybrid treatment with several hours separating the surgical and percutaneous procedures.

Since our Institution has already experience in staged hybrid treatment, a one-stage hybrid treatment study was designed to evaluate the feasibility of a one-stage approach for complex treatment of multivessel coronary artery disease.

## Aims

To determine the efficacy of one-stage hybrid revascularization in a long-term follow-up and evaluate the aspects of the periprocedural protocol.

## Materials and methods

### Protocol

This is a prospective, single-center feasibility study. The approval of the local bioethical committee was obtained (Beskidzka Izba Lekarska w Bielsku-Białej, approval no. 2013/02/07/2). The study was conducted according to the Declaration of Helsinki. Participation was offered to patients who were eligible for the study (based on inclusion and exclusion criteria and the possibility to perform both stages of the procedure, which was confirmed by a heart-team assessment—see below). All of them have agreed to participate in the study and signed the informed consent prior to study procedures.

### Patients

The inclusion criteria were: multivessel coronary artery disease, significant coronary artery stenosis (stenosis > 70%; stenosis with related symptoms confirmed in non-invasive tests; FFR ≤ 0.80 or minimal artery diameter ≤ 4.0 mm^2^), stable coronary disease, or acute coronary syndrome with no enzyme elevation (unstable angina).

The exclusion criteria were: previous percutaneous interventions, previous coronary artery bypass grafting, participation in any clinical study, pregnancy, planned surgical procedure (6 months after the hybrid treatment), contraindications to dual antiplatelet therapy, ejection fraction < 30%, creatinine clearance < 30 ml/min/1.73 m^2^, platelet count <100,000/mm^3^ or >700,000/mm^3^, cerebrovascular incident (including TIA) 6 months before the hybrid procedure, active gastrointestinal bleeding, life expectancy <1 year. In each case, the heart team assessed the validity and possibility of performing both parts of the hybrid procedure in accordance with clinical guidelines and anatomical features of coronary arteries.

### Procedures

On their admission to the hospital, informed consent was obtained, and physical examination, electrocardiography, echocardiography, and lab test panel (electrolytes, blood morphology, creatinine, creatine kinase- MB isoenzyme, high sensitivity troponin T, indexed normalized ratio of prothrombin time, and activated partial thromboplastin time) were conducted. The P2Y12 inhibitor was given pre-operatively or intra-operatively through a nasogastric tube, but always before the percutaneous part of the procedure.

A hybrid procedure was performed in the fully equipped hybrid operating room with C-arm. General mixed anesthesia was induced. An invasive arterial blood pressure monitoring (preferably right radial artery) was obtained. The intubation was performed with the use of a double-lumen endotracheal tube. A central venous port was introduced through the right jugular vein. A Foley catheter was passed into the bladder.

Endoscopic atraumatic coronary artery bypass grafting (EACAB) was performed first. Single, right lung ventilation was used for the surgical stage. The 3rd (anterior axillary line), 5th (medial axillary line), and 7th (anterior axillary line) intercostal spaces were used for endoscopic port insertion. Port in the 5th intercostal space was used for endoscopic camera insertion, and ports in the 3rd and 7th intercostal space were used for harmonic blade and laparoscopic forceps insertion. The internal thoracic artery (LITA) was harvested with the use of a harmonic blade (Ethicon, Bridgewater, NJ, USA) under endoscopic vision (Karl Storz, Tuttlingen, Germany).

Before LITA clipping, heparin was given in a dose of 1.5 mg/kg. Target activated clotting time (ACT) was 200–300 s. Left anterolateral mini-thoracotomy (4th intercostal space) was made for left anterior descending (LAD) exposure. The LITA-LAD anastomosis was made using a continuous 8.0 Prolene suture (Ethicon, NJ, USA). If not given pre-operatively, the ADP inhibitor Ticagrelor in a loading dose (180 mg) was administered through a nasogastric tube by the time of anastomosis completion and was continued at least for 12 months.

The ACT was monitored prior to percutaneous intervention to avoid unnecessary repeat heparin administration. Non-LAD vessels were stented with contemporary drug-eluting stents through femoral access. The angiography of LITA-LAD anastomosis was performed in every case. Protamine sulfate was not administered routinely.

In case of any doubts regarding the proper function of the LITA-LAD bypass graft, the anastomosis would be surgically revised during the same procedure and re-evaluated to verify the success of the procedure.

### Hospitalization

Constant invasive blood pressure, saturation, ECG, diuresis, and drainage monitoring were conducted for 48 h following the procedure. Dual antiplatelet therapy was maintained from the first day after the procedure with the intention to continue for 1 year. A chest x-ray was done after the surgery and 24 h after the surgery, when the chest tube was removed. The control echocardiography was performed 48 h after the procedure and whenever it was indicated in accordance with the patient’s clinical status. The patients were discharged to the rehabilitation department for rehabilitation and 30-day observation.

### Follow-up observation

The follow-up visits were scheduled for 30 days, 12 months, and yearly after the procedure. To fulfill the purpose of this report, all the patients had at least 12 months of follow-up completed (completion for 12-month follow-up was 100%). The phone call to each patient was scheduled for 6 months after the procedure. Dual antiplatelet therapy was indicated for 12 months.

For adverse events related to treated arteries during follow-up, heart-team consultation was conducted to choose the most optimal treatment in each case.

### Primary endpoints

Primary endpoints were all-cause mortality and composite primary endpoint—MACCE (major adverse cardiac and cerebrovascular events), defined as cardiac death, myocardial infarction, repeat revascularization, and stroke.

### Secondary endpoints

Periprocedural transfusions (>2 units of packed red blood cells), bleeding incident (TIMI scale), kidney injury, and arrhythmias (other than AF) were reported.

### Statistical analysis

Continuous data are presented as median (interquartile range). Categorical data are presented as a number (percentage). Shapiro–Wilk test was used to verify normal distribution in analyzed parameters. As normal distribution was rejected, the Mann–Whitney *U*-test was used for continuous data analysis. The Chi-square test was used for categorical data analysis. Kaplan–Meier curves were drawn for mortality and MACCE long-term evaluation. The data were analyzed using MedCalc v.18.5 (MedCalc Software, Ostend, Belgium).

## Results

The heart team carefully evaluated each referred case with the intention to select subjects who would probably benefit most from such procedures in accordance with complex inclusion and exclusion criteria, as well as clinical and anatomical features of the disease. Consequently, all procedures were completed in a 4-year timeframe.

The study population included 30 patients, similar in terms of comorbidities and perioperative risk. The echocardiography revealed no valvular disease or severe global contractility impairment (Tables 1, 2).

**TABLE 1:** Baseline patient characteristics.

Patients population	N = 30
Male	21 (68%)
Female	9 (32%)
Age (years)	63.0 (61.17–64.82)
Chronic coronary syndrome	8 (26.7%)
Unstable angina	22 (73.3%)
History of myocardial infarction	8 (26.6%)
Unsuccessful PCI LAD	1 (3.3%)
Two-vessel CAD	26 (86.7%)
Three-vessel CAD	4 (13.3%)
Diabetes t. 2	10 (33.3%)
Insulin therapy	5 (16.6%)
Arterial hypertension	28 (93.3%)
Hypercholesterolemia	26 (86.6%)
Smoking	8 (26.6%)
Asthma/COPD	2 (6.6%)
History of stroke/TIA	2 (6.6%)
Atrial fibrillation	1 (3.3%)
Obesity	16 (53.3%)
BMI (kg/m^2^)	30.3 (25.15–34.45)
Syntax score	28.0 (25.0–33.5)
Euroscore II (%)	0.98 (0.70–1.08)

Data are presented as median (interquartile range) and number (percentage). BMI, body mass index; CAD, coronary artery disease; COPD, chronic obstructive pulmonary disease; LAD, left anterior descending artery; PCI, percutaneous intervention; TIA, transient ischemic attack.

**TABLE 2:** Pre-operative echocardiography.

Echocardiographic parameters	N = 30
EF (%)	55.0 (50.0–55.25)
LA (mm)	38.5 (35.0–43.0)
LV ESD (mm)	35.0 (33.25–38.25)
LV EDD (mm)	51.0 (50.0–55.0)
PW (mm)	11.0 (11.0–11.25)
IVS (mm)	11.0 (10.75–12.0)
RV (mm)	26.0 (24.0–29.0)

Data are presented as median (interquartile range). EF, ejection fraction; IVS, intraventricular septum; LA, left atrium; LV EDD, left ventricular end-diastolic diameter; LV ESD, left ventricular end-systolic diameter; PW, posterior wall; RV, right ventricle.

Patients underwent successful EACAB procedures with LITA-LAD anastomosis. Ticagrelor was used as a P2Y12 antagonist in all but one case (Table 3). The LITA-LAD graft was evaluated during the percutaneous part of the procedure, showing that the surgery was successful in all patients.

**TABLE 3:** The one-stage hybrid procedure.

Hybrid procedure	N = 30
LITA-LAD	30 (100%)
Ticagrelor (loading dose)	29 (96.6%)
Clopidogrel (loading dose)	1 (3.3%)
Acetylsalicylic acid	100%
PCI ¿ 1 artery	4 (13.3%)
**PCI lesion type (34 lesions treated):**	
LCx/OM	19
IM	1
RCA	14
A *n* (%)	10 (29.4%)
B1	11 (32.3%)
B2	10 (29.4%)
C	3 (8.9%)
**EACAB lesion type (30 lesions treated):**	
A *n* (%)	2 (6%)
B1	4 (14%)
B2	6 (20%)
C	18 (60%)
EES (Xience, Promus, Synergy)	30 (88.2%)
ZES (Resolute)	4 (11.8%)
Median number of implanted DES	1.0 (1.0–2.0)
Radiation dose (mGy)	655.0 (442.7–1047.5)
Fluoroscopy time (min)	9.7 (7.9–14.2)
Contrast dose (ml)	150.0 (150.0–200.0)

Data are presented as number (percentage) and mean ± standard deviation. DES, drug eluting stent; LITA-LAD, left internal mammary artery–left anterior descending artery bypass graft; PCI, percutaneous coronary intervention.

By the time of the 30-day follow-up, no death, stroke, or myocardial infarction were observed. One of the patients underwent coronarography and repeat LAD revascularization due to LITA-LAD failure. One patient required chest revision and transfusion of >2 PRBC due to surgical bleeding. Kidney injury, defined as 2 × creatinine raise (RIFLE classification), was noted in two patients (Table 4). Overall primary endpoint rate was 10%. The perioperative arrhythmia included only atrial fibrillation, which occurred in four cases and was successfully treated.

**TABLE 4:** 30-day observation toward primary endpoints.

Perioperative endpoints (up to 30 days)	N = 30
Death	0
Myocardial infarction	0
Repeat revascularization	0
Stent thrombosis	0
Stroke	0
Transfusions (>2 units of PRBC)	1 (3.3%)
Major CABG-related bleeding (TIMI)	1 (3.3%)
Kidney injury (RIFLE criteria)	2 (6.6%)
Arrhythmia (atrial fibrillation)	4 (13.3%)

The data are presented as a number (percentage). RIFLE, risk, injury, failure, loss of function, end stage disease classification for kidney injury; TIMI, thrombosis in myocardial infarction bleeding scale.

**FIGURE 1:**
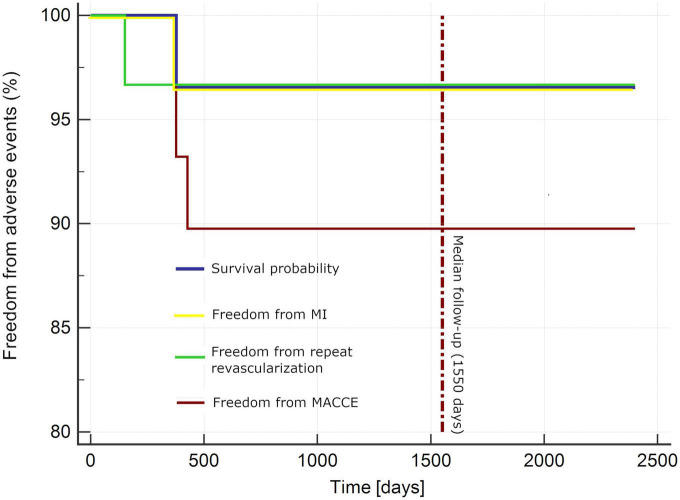
Kaplan–Meier curve for adverse events. MACCE, major adverse cardiac and cerebrovascular incidents (death, myocardial infarction, stroke, repeat revascularization); MI, myocardial infarction.

Table 5 and Figure 1 present extended observations toward MACCE (primary endpoints). One patient underwent repeat percutaneous revascularization in LAD (significant stenosis at LITA-LAD anastomosis site) and repeat stenting of previously PCI-treated vessels. Another subject underwent percutaneous revascularization in a previously untreated vessels 1 year following the hybrid procedure (the stenosis was insignificant by the time of hybrid treatment). Furthermore, one patient died from sudden cardiac death related to myocardial infarction 1 year following the procedure (Table 5 and Figure 1). No other incidents of myocardial infarction were reported.

**TABLE 5:** Long-term observation toward MACCE.

Median long term follow-up (years)	4.25 (2.62–4.69)
Follow-up completion	30 (100%)
Overall MACCE (composite endpoint)	3 (9.9%)
Death	1 (3.3%)
Myocardial infarction	1 (3.3%)
Repeat revascularization (LAD)	1 (3.3%)
Repeat revascularization (non-LAD)–PCI treated arteries	1 (3.3%)
Revascularization of previously untreated arteries	1 (3.3%)
Stroke	0

The data are presented as a number (percentage) and median (interquartile range). LAD, left anterior descending artery; MACCE, major adverse cardiac and cerebrovascular incidents (death, myocardial infarction, repeat revascularization, stroke).

The observation of other perioperative parameters is summarized in Table 6.

**TABLE 6:** Perioperative parameters.

Perioperative	N = 30
Total drainage (ml)	750 (422.5–1112.5)
Chest revision	1 (3.3%)
Inotrope use	4 (13.3%)
Ventilation time (min)	430.0 (300.0–600.0)
PRBC transfusion	8 (26.6%)
>2 units of PRBC	1 (3.3%)
FFP transfusion	5 (16.6%)
Platelet transfusion	1 (3.3%)
Pleurocentesis	11 (36.6%)
CVVHDF	1 (3.3%)
EF (%)	55.0 (50.0–56.50)
CK-MB 24 h (ng/ml) (*N*: <7,2)	8.7 (5.7–14.9)
CK-MB 48 h (ng/ml) (*N*: <7,2)	3.8 (3.2–5.4)
Troponin T high sensitivity 24 h (pg/ml) (*N*: 0–14)	46.7 (32.3–68.6)
Troponin T high sensitivity 48 h (pg/ml) (*N*: 0–14)	44.8 (23.1–75.5)

Data are presented as number (percentage) and median (interquartile range). CK-MB, creatine kinase (MB isoenzyme); CVVHDF, continuous veno-venous hemodiafiltration; EF, ejection fraction; PRBC, packed red blood cells.

Although the study was not powered for that purpose, some observations may be gathered from the P2Y12 inhibitor administration protocol. Eight patients received P2Y12 inhibitor intra-operatively (nasogastric tube) and 22 subjects were given the medication before the procedure. No impact on both thrombotic and bleeding complications was observed (Table 7).

**TABLE 7:** Comparison of preprocedural and intra-operative P2Y12 inhibitor administration subgroups.

	Pre-procedural P2Y12 inhibitor	Intra-operative P2Y12 inhibitor	*p*
Number of patients	22 (73%)	8 (27%)	*P* < 0.001
Stent thrombosis	0	0	1
Drainage	522.5 (350.0–800.0)	640.0 (352.5–1000.0)	0.542
Blood transfusion	4 (18%)	2 (25%)	0.645
Pleurocentesis	6 (27%)	2 (25%)	1

Data are presented as number (percentage) and median (interquartile range).

## Discussion

Our report provides valuable information regarding one-stage hybrid coronary revascularization, indicating that even with the use of concomitant dual antiplatelet therapy during surgical procedures, it is a safe and efficient method of achieving the complete clinical effect in selected patients. The follow-up, which is completed in 100%, is one of the longest in similar studies and clearly demonstrates outstanding long-term results in this group of patients.

Hybrid coronary revascularization is a promising method, often described as “the best of two worlds.” It combines the advanced, mini-invasive approach with an assumption of achieving satisfactory long-term results. However, the evidence is not complete. Randomized trials with hybrid revascularization as a treatment are limited and often underpowered.

Despite this fact, hybrid revascularization is believed to be an interesting option for multivessel coronary disease treatment. Its importance has been growing in successive guideline releases (1). The interest is well justified as both left internal thoracic artery and new generation drug-eluting stents have certainly met their therapeutic expectations.

The LITA-LAD graft is associated with superior long-term patency compared to PCI (6) and the long-term results of EACAB surgery are outstanding (7). Furthermore, the LITA-LAD grafting reduces the need for future revascularization in the non-LAD vessels while providing long-term relief from anginal episodes (8).

Additionally, saphenous vein grafts have limited patency and may be inferior to new generation drug eluting stents (6, 9). The risk of various wound complications associated with the sternotomy is estimated at 0.4–8% (10). A minimally invasive approach may reduce morbidity, pain, scarring, and recovery time when compared to sternotomy bypass grafting (11–13).

Consequently, the interest in hybrid coronary revascularization is constantly growing and many investigators seek optimal treatment protocols. Two-stage coronary revascularization ensures satisfactory results and does not require a hybrid operating room. However, it may be associated with some disadvantages. The MIDCAB-first patients have a risk of ischemia originating from non-LAD territories during the LITA-LAD grafting and a risk of repeat surgical reintervention in case of an unsuccessful PCI. On the other hand, PCI-first patients have an aggravated risk of stent thrombosis, increased perioperative bleeding due to dual antiplatelet therapy, and adverse events in the LAD territory during the separating time interval (14). Furthermore, LITA-LAD anastomosis is not evaluated in angiography in the PCI-first strategy.

Most of those issues are addressed in a one-stage hybrid procedure protocol. The procedure requires a hybrid operating room and the entire operating team must be experienced to achieve optimal clinical outcomes. The one-stage HCR ensures that the PCI to high-risk non-LAD lesions can be performed with a protected LAD, conventional CABG remains an option in cases of unsuccessful stent implantation and LITA-LAD graft can be studied before PCI. Furthermore, it is cost-effective, as it reduces hospital length of stay and patients remain more satisfied if the revascularization therapy is completed in one encounter.

The evidence of a one-stage strategy is limited, as only several centers have fully equipped hybrid operating rooms. However, it may be expected that the number will be increasing. Few reports exist and there are no adequately powered randomized trials that could be used for specific treatment protocols. Some experts point to disadvantages of one-stage treatment—the inflammatory response to surgery results in a risk for stent thrombosis, dual antiplatelet therapy increases the risk of bleeding, and the patients are exposed to the dual nephrotoxic insult of surgery and contrast media utilization (14).

The current study was designed to address those doubts. We did not observe any acute coronary syndrome or acute stent thrombosis in the perioperative period. Both options of administering ticagrelor (pre-operative and through the nasogastric tube when the surgical part of the procedure is nearly completed) resulted in safe lesion stenting and did not have a negative impact on hemostasis following the surgical part of the procedure.

No stroke was observed in the entire group. It may be important to extend the analysis, as stroke following coronary artery bypass grafting is one of the most devastating complications, entailing permanent disability and a 3–6-fold increased risk of death with a case-fatality rate of up to 20% (15, 16). The risk of stroke after CABG varies across studies ranging from 0.0 to 5.2%, depending on study design, patient risk profile, operative techniques, and the length of study follow-up (17, 18). Hopingly, the absence of cardiopulmonary bypass and aortic manipulation may reduce the risk of cerebrovascular incidents while achieving complete revascularization.

Surgical procedures usually require 24-h drainage observation. To evaluate the potential drawbacks of the one-stage strategy, careful evaluation of all bleeding incidents was required. TIMI classification and blood product requirements were chosen to study the potential issues in this matter. Both secondary endpoints (CABG-related bleeding with accordance to TIMI classification, transfusion > 2 PRBC) were noted only in one case. The patient required chest revision and assessment of bleeding from the anastomosis site. As such, this situation should be considered as rather a technical issue than a protocol drawback.

The endpoint referring to blood transfusions (>2 units of packed red blood cells) is well justified, as coronary angiography contributes to a 1.8 g/dL reduction in hemoglobin concentration before CABG surgery and was associated with increased post-operative transfusion of allogeneic blood products (19). Furthermore, a recent meta-analysis concludes that 22.8% of HCR patients received a blood transfusion compared with 46.1% of the CABG group (20). Our revascularization strategy did not exceed the values reported in the hybrid group.

The LITA-LAD anastomosis is considered to provide superior long-term outcomes. It has been shown to be more durable than other arterial and vein grafts as well as coronary stents for treatment of LAD disease, with patency rates > 90% at 5-year follow-up (6, 8, 21). When ITA graft failure occurs, a technical error is the most common cause in the early post-operative period. In the subsequent weeks to months, localized neointimal hyperplasia may occur at the cleft between the native artery and the ITA graft at the anastomotic suture site, on the hood, and on the floor of the native LAD, which can result in localized stenosis (22, 23). Consequently, we do not correlate the need for repeated LAD revascularization in one case with the treatment protocol.

Acute kidney injury associated with cardiac surgery procedures is the second most common cause of AKI in the intensive care setting (after sepsis) and is independently associated with increased morbidity and mortality. Furthermore, when AKIN or RIFLE criteria are applied, the mortality rate (hospital discharge or 30-day mortality) is between 3.8 and 54.4% in patients who develop CSA-AKI and increases progressively with the degree of renal impairment (24). Consequently, assessing the renal outcomes of the hybrid procedure was one of the key aspects of the study. Only two of our patients underwent kidney injury in accordance with the RIFLE criteria (>2 × creatinine raise). It must be noted that one of them suffered a kidney injury, which was related to other perioperative complications, including bleeding, reoperation, and massive transfusion. The same patient required temporary CVVHDF for hemodynamic and renal stabilization. Recent meta-analysis revealed a renal failure incidence of 1.7% in the HCR group, compared with 2.6% in the CABG group (20).

Atrial fibrillation was the only noted arrhythmia. The complication is very common after surgical procedures. It worsens the post-operative state and prognosis and increases the length of ICU stay, hospitalization, and hospital costs considerably (25, 26). Seven studies examined the incidence of post-operative atrial fibrillation- in the HCR group, the incidence of POAF was 17%, compared with 19.2% in the CABG group (20). In our study, we observe even fewer episodes of arrhythmia, which makes the protocol even more convincing.

Recently, results from a randomized trial comparing coronary artery bypass grafting, hybrid coronary revascularization, and multivessel percutaneous intervention were published. The authors report that residual myocardial ischemia and MACCE were similar at 12 months (27). The results are encouraging; however, they cannot be directly compared to our study due to the staged protocol of hybrid treatment.

The MERGING clinical trial provided late clinical outcomes of myocardial hybrid revascularization (28). In this study, the percutaneous phase was performed 48–72 h after the withdrawal of chest tubes, which makes the perioperative protocol much different from ours. However, the authors note that hybrid coronary revascularization was associated with increased rates of major adverse cardiovascular events during 2 years of clinical follow-up, including the main incidence of unplanned revascularization (14.5% in the HCR vs. 5.9% in the CABG group). Our study does not support this data; however, further investigation in a randomized clinical trial is indicated.

It is crucial to mention that the results of the “HYBRID-COR” feasibility study by the time of patient evaluation were superior to the reported 5-year Syntax trial outcome, regardless of revascularization strategy (mortality and MACCE in patients with a SYNTAX score of 23–32: 12.7 and 25.8% in CABG group; 13.8 and 36% in PCI group) (29).

### Study limitations

The study has its drawbacks: it is a single-center feasibility report, with no control group and the analyzed sample is relatively small. Furthermore, no routine coronary angiography was performed in asymptomatic patients during follow-up.

## Conclusion

From our initial experience, we can conclude that the single-stage coronary hybrid revascularization while on the double antiplatelet therapy is feasible and safe. It offers high procedural success and complete revascularization. The single-digit repeated revascularization rate and low MACCE rate at 4 years show excellent outcomes, better than previously reported PCI and CABG procedures alone. Further studies, including randomized clinical trials, are indicated.

## Data availability statement

The original contributions presented in this study are included in the article/supplementary material, further inquiries can be directed to the corresponding author.

## Ethics statement

The studies involving human participants were reviewed and approved by Beskidzka Izba Lekarska w Bielsku-Białej (approval no. 2013/02/07/2). The patients/participants provided their written informed consent to participate in this study.

## Author contributions

All authors listed have made a substantial, direct, and intellectual contribution to the work, and approved it for publication.
